# Non-vaccinal prophylaxis of tularemia

**DOI:** 10.3389/fmicb.2024.1507469

**Published:** 2024-11-28

**Authors:** Max Maurin, Aurélie Hennebique, Camille Brunet, Léa Pondérand, Isabelle Pelloux, Sandrine Boisset, Yvan Caspar

**Affiliations:** ^1^Centre Hospitalier Universitaire Grenoble Alpes, Centre National de Référence Francisella Tularensis, , Grenoble, France; ^2^Recherche Translationnelle et Innovation en Médecine et Complexité (TIMC), Centre National de la Recherche Scientifique (CNRS), Université Grenoble Alpes, Grenoble, France; ^3^Université Grenoble Alpes, Commissariat à l’énergie atomique (CEA), Centre National de la Recherche Scientifique (CNRS), Institut de Biologie Structurale (IBS), Grenoble, France

**Keywords:** tularemia, *Francisella tularensis*, prophylaxis, antibiotic prophylaxis, zoonosis, risk factors, occupational disease, arthropod-borne diseases

## Abstract

Tularemia is a re-emerging zoonosis in many endemic countries. It is caused by *Francisella tularensis*, a gram-negative bacterium and biological threat agent. Humans are infected from the wild animal reservoir, the environmental reservoir or by the bite of arthropod vectors. This infection occurs through the cutaneous, conjunctival, digestive or respiratory routes. Tularemia generally manifests itself as an infection at the site of entry of the bacteria with regional lymphadenopathy, or as a systemic disease, particularly pulmonary. It is often a debilitating condition due to persistent symptoms and sometimes a life-threatening condition. There is effective antibiotic treatment for this disease but no vaccine is currently available for humans or animals. Due to the complexity of the *F. tularensis* life cycle and multiple modes of human infection, non-vaccine prophylaxis of tularemia is complex and poorly defined. In this review, we summarize the various individual prophylactic measures available against tularemia based on the different risk factors associated with the disease. We also discuss the currently underdeveloped possibilities for collective prophylaxis. Prophylactic measures must be adapted in each tularemia endemic area according to the predominant modes of human and animal infection. They requires a One Health approach to control both animal and environmental reservoirs of *F. tularensis*, as well as arthropod vectors, to slow the current expansion of endemic areas of this disease in a context of climate change.

## 1 Introduction

Tularemia is a zoonosis caused by *Francisella tularensis* (Ellis et al., 2002). This gram-negative bacterium is a highly virulent biological threat agent (Dennis et al., 2001; Maurin, 2015). It can infect a broad range of vertebrate animals, primarily mammals, and arthropods such as ticks and mosquitoes (Telford and Goethert, 2020). In addition, it can survive for prolonged periods in aquatic and soil environments, usually contaminated from infected animals (Hennebique et al., 2019).

Human tularemia cases have been increasingly reported in most endemic countries during the last two decades (Erdem et al., 2014; Maurin and Gyuranecz, 2016; Plymoth et al., 2024; Wu et al., 2024). This is partly related to improved surveillance, diagnosis and reporting of human and animal tularemia cases owing to a renewed medical interest following the classification of *F. tularensis* as a biological threat agent. Tularemia is currently a notifiable disease in many countries and epidemiological surveillance of this disease by public health organizations has been strengthened. In addition, tularemia has emerged in Spain (Mínguez-González et al., 2021) and has been newly discovered in South Australia (Jackson et al., 2012).

Data for a vaccine prophylaxis to tularemia have been previously summarized (Conlan, 2011; Marohn and Barry, 2013; Jia and Horwitz, 2018). Virulence attenuated strains, such as the live vaccine strain (LVS), have been used in the past for tularemia prophylaxis in the general population or in laboratory staff handling *F. tularensis* cultures (Conlan, 2011). However, these vaccines afford minimal protection against the most severed forms of tularemia. In addition, because the mechanism of virulence attenuation has not been fully characterized, there is a concern about the possibility of reversion of the vaccine strains to a fully virulent state. Many innovative tularemia vaccines have been developed in recent years, but none is currently approved for human or animal use (Conlan, 2011; Marohn and Barry, 2013; Jia and Horwitz, 2018).

This review aims to emphasize literature data dealing with a non-vaccinal prophylaxis for tularemia in endemic areas. We have summarized data on risk assessment of exposure to *F. tularensis* according to the geographic areas and populations considered. We have discussed data on post-exposure antibiotic prophylaxis including its basic principles and current recommendations. We have presented and discussed the individual primary prophylaxis measures currently recommended but also proposed non-vaccine collective prophylactic measures that seem essential for the control of tularemia, in a One Health approach.

## 2 Search strategy and selection criteria

Data were collected from English literature in the PUBMED database using the following keywords: *F. tularensis* or tularemia, and one of the following terms, prophylaxis, antibiotic prophylaxis, prevention, epidemiology, arthropods, mosquitoes, mosquito-borne, ticks, tickborne, occupation, occupational diseases, animal models, human prophylaxis, food, foodborne, environment, laboratory infections, and healthcare workers. Some reviews on tularemia or *F. tularensis* were included. In total, 174 articles were selected for this review.

## 3 Francisella tularensis

### 3.1 Taxonomy and virulence

*Francisella tularensis* is divided in three subspecies, two of which are responsible for tularemia (Ellis et al., 2002). *F. tularensis* subsp. *tularensis* (also referred as type A), the most virulent subspecies, is restricted to North America. *F. tularensis* subsp. *holarctica* (type B) is found in the whole northern hemisphere and southern Australia (Jackson et al., 2012). Although *F. tularensis* is a monophyletic species with a highly conserved genome, molecular methods (e.g., MLVA and canonical SNPs analysis) have allowed defining specific clades and subclades (Öhrman et al., 2021). Major clades are A1 and A2 for type A strains, and B4, B6, B12, and B16 for type B strains. These clades have wide but variable geographic distributions that can overlap. Virulence variations have been reported between type A strains clades and subclades (Kugeler et al., 2009).

*Francisella tularensis* is one of the most virulent bacterium in humans (Degabriel et al., 2023). This bacterium resists the killing effects of complement, antibodies, and cationic antimicrobial peptides owing to the presence of a capsule and an unusual lipopolysaccharide (LPS) structure. This bacterium also resists the adapted immune response due to its ability to replicate inside phagocytic cells. *F. tularensis* has a specific LPS (particularly a lipid A which is tetra-acylated, with long acyl chains, and hypophosphorylated) that is not recognized by the Toll-like receptor 4 (TLR4) and only triggers limited TLR2-mediated innate immune responses. After engulfment by macrophages or dendritic cells, *F. tularensis* escapes from its phagosomal vacuole to replicate in the eukaryotic cell cytosol. Genes clustered in the *F. tularensis* pathogenicity island (FPI) encode a type 6 secretion system that allows the bacteria to lyse the phagosomal membrane and reach the cell cytosol. Phagosomal escape is also promoted by bacterial synthesis of biotin, enzymes (e.g., arginine permease), and ammonia to alkalinize the acidic phagosome. Within the nutrient-rich cell cytosol, metabolic adaptations allow *F. tularensis* to replicate using host cell growth factors (e.g., amino acids). Cell to cell spread can result from lysis of infected cells or merocytophagy. Although the immune system is usually able to control *F. tularensis* infection, an acute infection can overwhelm the immune system’s response capabilities and become life-threatening.

### 3.2 *Francisella tularensis* reservoirs and arthropod vectors

#### 3.2.1 Animal reservoir

*Francisella tularensis* has been detected in many animal species, including mammals, birds, reptiles, amphibians, fish, and some invertebrate species (Telford and Goethert, 2020). Lagomorphs and small rodents are considered primary sources of human infections. Tularemia transmission within wildlife likely occurs by direct animal–animal contact, from the contaminated environment, and via arthropod vectors (Carvalho et al., 2014; Sharma et al., 2023). The presentation and severity of tularemia can vary significantly depending on the animal species affected (Carvalho et al., 2014; Sharma et al., 2023). Similar to humans, infections caused by type A strains tend to be more severe than those from type B strains. In animals infected with *F. tularensis*, symptoms can differ based on the route of infection, whether respiratory, digestive, or cutaneous. However, these symptoms are generally nonspecific and may include fever, ruffled fur, anorexia, depression, coughing, vomiting, diarrhea, conjunctivitis, ataxia, lethargy, and prostration. Clinical examination may reveal signs such as fever, dehydration, weight loss, ulceration of the tongue and oropharynx, jaundice, enlarged lymph nodes, draining abscesses, splenomegaly, and hepatomegaly. Pathological findings in animals that succumb to tularemia may include tracheitis, bronchitis, pneumonia, splenomegaly, hepatomegaly, and either regional or generalized lymphadenopathy. Additionally, various organs may exhibit congestive, nodular, hemorrhagic, or necrotic lesions. Although difficult to assess for natural infections, tularemia severity greatly vary among animal species (Carvalho et al., 2014; Sharma et al., 2023). Most bird species are believed to have a natural resistance to *F. tularensis* infection. In contrast, domestic animals such as sheep, pigs, and horses are susceptible to tularemia. Lagomorphs, many small rodent species, cats, and dogs can experience severe and occasionally fatal infections. However, susceptibility to tularemia varies within lagomorph and rodent species, and cats usually develop more severe disease than dogs.

#### 3.2.2 Arthropod vectors

Several arthropod species are capable of transmitting *F. tularensis* within the animal reservoir and to humans (Petersen et al., 2009; Telford and Goethert, 2020).

##### 3.2.2.1 Ticks

Tick species involved in *F. tularensis* transmission vary according to geographic areas, but include *Ixodes*, *Dermacentor*, and *Amblyomma* species (Telford and Goethert, 2020). Transstadial transmission of *F. tularensis* occurs in ticks, allowing them to harbor the bacterium throughout their life cycle. Tick larvae that become infected after feeding on an infected animal host can transmit *F. tularensis* during subsequent life stages, when they molt into nymphs and adults. Given that ticks can live for several years, they serve as a significant reservoir for *F. tularensis* (Telford and Goethert, 2020). Transovarial transmission of this bacterium has not been formally demonstrated. The prevalence of *F. tularensis* infection in ticks can be determined by PCR techniques, which must specifically amplify DNA from this species and not that of *Francisella*-like tick endosymbionts (Kugeler et al., 2005; Escudero et al., 2008). In most reported studies, ticks were tested in pools and only the percentages of *F. tularensis-*positive pools were determined. Thus, results were expressed as minimum infection rate (MIR), considering only one positive tick per positive pool. Although likely underestimated, the *F. tularensis* tick infection rates were overall low but varied according to the geographic areas. As examples, MIR were 0.27% for 5,402 ticks from Hungary (Kreizinger et al., 2013), 0.89% for 2,134 ticks from Spain (Lopes de Carvalho et al., 2016), 1.2% for 4,197 ticks from Iran (Esmaeili et al., 2023), 0.45–3.45% for 1,551 ticks from Poland (Bielawska-Drózd et al., 2018), 3.6% for more than 3,000 ticks from Minnesota in the USA (Whitten et al., 2019), and 8.4% for 916 *Ixodes ricinus* ticks from Baden-Wuerttemberg federal state of Germany (Gehringer et al., 2013).

##### 3.2.2.2 Mosquitoes

Mosquito-borne tularemia is restricted to specific geographic areas, including Sweden and Finland (Abdellahoum et al., 2020; Telford and Goethert, 2020). Therefore, only *F. tularensis* subsp. *holarctica* has been associated with this mode of transmission. Several mosquito species can be vectors of *F. tularensis.* In Sweden, natural *F. tularensis* infection has been detected in *Aedes cinereus*, *Ae. vexan*, *Ae. sticticus*, *Ae. annulipes*, *Ae. intrudens*, *Ae. leucomelas*, *Ae. cantans*, *Anopheles claviger*, *An. maculipennis*, *Coquillettidia richiardii*, and *Culex pipiens*/*torrentium* (Lundström et al., 2011; Thelaus et al., 2014; Dryselius et al., 2019). It is believed that mosquitoes can become infected during their larval stage in aquatic environments contaminated with *F. tularensis*. Then, the bacteria are transmitted transstadially through the different larval stages, then to pupae and adult mosquitoes. Transovarial transmission of *F. tularensis* from female mosquitoes to their offspring has not been formally demonstrated. A few studies have evaluated *F. tularensis* infection prevalence in mosquitoes. A high prevalence was reported among 14,267 mosquitoes collected in Örebro, an endemic areas of Sweden, with 36 positive mosquito pools among 277 studied (i.e., 12.9%), representing 11 mosquito species among 14 evaluated (Thelaus et al., 2014). These species belonged to the *Aedes*, *Anopheles*, *Coquillettidia*, and *Culex* genera. The same authors reported a 25% rate of transmission of *F. tularensis* from experimentally infected larvae to adults (Thelaus et al., 2014).

##### 3.2.2.3 Other arthropods

Dear flies (*Chrysops*, *Tabanidae* family) have been associated with *F. tularensis* transmission to humans in few cases and mainly in Utah in the USA (Calanan et al., 2010). These flies are considered passive vectors of *F. tularensis*, i.e., they do not support multiplication of this bacterium in their bodies but transmit it through their mouthparts. Fleas, lice, bedbugs, and mites have been experimentally infected with *F. tularensis* but are currently not considered natural tularemia vectors for humans (Telford and Goethert, 2020).

#### 3.2.3 Environment

Field studies have shown that *F. tularensis* is widespread in soil, fresh water, and brackish water, although its isolation from environment samples has been rarely obtained (Kaysser et al., 2008; Broman et al., 2011; Simşek et al., 2012; Janse et al., 2018; Brunet et al., 2021). *F. tularensis*-infected animals are likely the primary sources of environmental contamination through their feces, urine, and carcasses. *In vitro*, *F. tularensis* can remain viable for several months in water without adding nutrients, at variable temperature (~4°C-20°C) and salinity (0–10 mg/L) (Forsman et al., 2000; Golovliov et al., 2021; Brunet et al., 2022; Cantlay et al., 2024). Long-term survival of *F. tularensis* in aquatic environments is likely related to its ability to evolve to a viable but non-culturable (VBNC) state, interact with protozoa such as amoebae, and form biofilms (Abd et al., 2003; Buse et al., 2016; Ozanic et al., 2016; Hennebique et al., 2021; Schaudinn et al., 2023).

## 4 Tularemia

### 4.1 Endemic areas

Tularemia is found in Northern America, including the United States and Canada. In USA, the global incidence of human tularemia between 2011 and 2020 (CDC) was 0.05 cases per 100,0000 residents (https://www.cdc.gov/tularemia/statistics/index.html#print, accessed January 25, 2024). The disease predominates in the central states such as Arkansas, South Dakota, Wyoming, Kansas, Nebraska, Missouri, and Oklahoma. High tularemia incidences are also reported in eastern and western USA. In Europe, the global incidence of tularemia was 0.14 cases per 100,000 people in 2022 (European Food Safety Authority (EFSA) and European Centre for Disease Prevention and Control (ECDC), 2023). Most cases occurred in Sweden and Finland. Mosquito-borne tularemia outbreaks occur almost annually in Sweden and less frequently in Finland (Rossow et al., 2014; Dryselius et al., 2019). Large outbreaks have been reported in the early 2000s in Spain (Pérez-Castrillón et al., 2001). In Asia, human tularemia cases predominate in Turkey, Japan, and China. Since the 2000s, several large outbreaks of water-borne tularemia have occurred in Turkey (Erdem et al., 2014). Oceania was considered free of tularemia for decades, but a few human infections related to possum bites were reported in 2012 in Tasmania, Australia (Jackson et al., 2012). The presence of tularemia in Africa has not been formally demonstrated.

### 4.2 Modes of contamination with *F. tularensis* in humans

Because *F. tularensis* has a large reservoir, the sources and modes of human infection are varied (Sjöstedt, 2007; Nelson and Sjöstedt, 2024). The primary route of infection is the skin in most tularemia endemic countries, including through direct contact with animals, arthropod bites, and direct contact with a contaminated environment (Liles and Burger, 1993; Dryselius et al., 2019; Hennebique et al., 2019; Kwit et al., 2019; Zellner and Huntley, 2019). The oral route of contamination correspond to the ingestion of contaminated food or water (Djordjevic-Spasic et al., 2011; Erdem et al., 2014; Burckhardt et al., 2018). Infections through the respiratory route occur when inhaling *F. tularensis* aerosols (Dahlstrand et al., 1971; Syrjälä et al., 1985; Feldman et al., 2003). *F. tularensis* infection also occur through the conjunctiva through handheld transmission or eye projections (Eren Gok et al., 2014; Lakos et al., 2020; Copur and Surme, 2023).

### 4.3 Tularemia incidence variations and seasonality

In most endemic areas, human tularemia cases occur throughout the year. However, according to the sources and modes of infection, tularemia may have a seasonality pattern. Human infections occurring after contact with game predominate during the hunting season (autumn and winter) (Jacob et al., 2020). Arthropod-borne tularemia cases predominate during the peak of activity of ticks and mosquitoes and when many people have outdoor activities (spring, summer, and autumn) (Bishop et al., 2023). Tularemia cases related to the consumption of *F. tularensis*-contaminated water have been reported to predominate in autumn and winter (Kilic et al., 2015).

The incidence of human infections has varied over time in many tularemia endemic areas. These variations are often related to changes in the population density of *F. tularensis*-carrying animals. Epizootics occurring in lagomorphs and small rodents have been reported to lead to an increased incidence of tularemia in humans living in the same geographic areas (Carvalho et al., 2014). However, a link between tularemia incidence variations in animals and in humans remains difficult to establish, particularly because the animal reservoir of *F. tularensis* remains poorly defined.

Human tularemia is usually a sporadic disease, with occasional small outbreaks, e.g., family outbreaks of food-borne infections (Greco and Ninu, 1985; Mailles and Vaillant, 2014). Large outbreaks can occur in countries and regions where human infections are related to mosquito bites or the consumption of contaminated water. In Sweden, epidemics involving hundreds of mosquito-borne infections have occurred almost annually since 2000 (Desvars et al., 2015). In Turkey, water-borne outbreaks have been reported in the last two decades in regions where people have limited access to potable water (Kilic et al., 2015). Large outbreaks occurred in Spain in the early 2000s, when tularemia emerged in this previously non-endemic country (Pérez-Castrillón et al., 2001).

### 4.4 Clinical manifestations

People infected with *F. tularensis* usually develop symptoms a few days later (usually 3–5 days, up to two weeks) (Tärnvik and Chu, 2007; Hepburn and Simpson, 2008; Erdem et al., 2014; Maurin and Gyuranecz, 2016; Darmon-Curti et al., 2020; Wu et al., 2024). They may develop severe symptoms (often with *F. tularensis* bacteremia) when infected with a type A strain or because of an immunocompromised status. Most people develop infections of mild to moderate severity. Generally, the disease manifests by flu-like symptoms such as fever, fatigue, cough, headache, arthralgia, and myalgia. Then, six clinical forms are classically recognized corresponding to different routes of infection. The ulceroglandular form, the most typical, combines a skin inoculation lesion with satellite regional lymphadenopathy. The glandular form is a regional lymphadenopathy without detectable inoculation lesion. The oculoglandular forms is a conjunctivitis with preauricular or cervical lymphadenopathy (i.e., the Parinaud’s oculoglandular syndrome). The oropharyngeal form is a pharyngotonsillitis with submandibular or cervical lymphadenopathy. The pneumonic form can be an acute or subacute pneumonia, or a chronic lung infection. This later presentation is particularly frequent with type B strains in Europe and Asia. Diagnosis is often delayed in patients with altered general status, weight loss, intermittent fever, moderate respiratory symptoms, and mediastinal or hilar lymphadenopathy on radiological exams (Martinet et al., 2021; Widerström et al., 2024). A high fever, usually with confusion but no detectable inoculation lesion or regional infection correspond to the typhoidal form. *F. tularensis* infection may lead to inaugural or secondary complications involving almost any organs. Lymph node suppuration occurs in about 30% of patients with lymphadenopathy. Other complications include meningitis and meningoencephalitis, aortitis, osteoarticular infections, intra-abdominal infections, and skin and soft tissue infections.

### 4.5 Variations according to age, gender, and underlying health condition

Tularemia cases related to contact with animals, tick bites, or a contaminated environment usually predominate in middle-aged adult males, likely because of more frequent work or leisure outdoor activities (Darmon-Curti et al., 2020; Wu et al., 2024). Tularemia cases related to mosquito bites or the consumption of non-potable water usually occur in the whole adult and pediatric population (Dryselius et al., 2019; Plymoth et al., 2024). The risk of *F. tularensis* exposure likely does not vary depending on the underlying health status of individuals. However, infections occurring in pregnant women can lead to obstetric complications (Ata et al., 2013) and those occurring in immunocompromised patients are often systemic and of the pneumonic form (Bahuaud et al., 2021).

## 5 Tularemia of direct animal sources: risk factors and prophylaxis

Some occupations involve frequent contact with live animals. Tularemia is a rare disease in livestock, and most frequently involves sheep (Jellison and Kohls, 1955; O’Toole et al., 2008). Farmers are considered at risk of tularemia but are exposed to multiple *F. tularensis* sources and rarely infected from farm animals (Jellison and Kohls, 1955). Veterinarians and their staff can acquire tularemia through handling animals, animal bites or scratches, or contact with animal body fluids (e.g., during surgery) (Liles and Burger, 1993; Büyük et al., 2016; Marx et al., 2024). Other occupations such as pet sellers and animal keepers are also exposed to this zoonotic risk. Human infection often occur through handling wildlife animals or their carcasses, or less frequently via bites or scratches from these animals. Forestry workers, game wardens, forest guards are occupations exposed to the wildlife fauna. Taxidermists and tanners can be exposed to animals dead from tularemia. Zoological park employees have been contaminated from zoo animals (Preiksaitis et al., 1979).

Individuals participating in recreational activities that involve contact with or bites from wild animals are at an elevated risk of contracting tularemia (Sjöstedt, 2007). Since tularemia is often fatal in many animal species, handling animal carcasses in the wild is particularly risky (Rossow et al., 2014). High-risk hobbies include hunting and trapping. However, few studies have evaluated the relative risk of tularemia in these populations compared to the general population. In endemic western regions of Germany, a tularemia seroprevalence of 1.7% was reported in 286 hunters compared to 0.2% in 6883 people of the general population (OR = 7.7, *p* < 0.001) (Jenzora et al., 2008). In the south-eastern Austrian federal states of Styria and Burgenland, five of 149 (3.35%) hunters displayed antibodies against *F. tularensis*, while none of 50 urban people had such antibodies. A tularemia seroprevalence of 6.3% (4/64) was reported in hunters in Yozgat province, in Central Anatolia region of Turkey, although two hunters developed oropharyngeal tularemia suggesting a water-borne infection (Yeşilyurt et al., 2012). In Quebec (Canada), a tularemia seroprevalence of 2.4% was reported in trappers compared to 0.6% in controls (Lévesque et al., 1995). Some wild animals have transmitted tularemia through bites or scratches to people walking outdoors, including squirrels, buzzards, a coyote, an hamster, and a dormouse (Magee et al., 1989; Centers for Disease Control and Prevention (CDC), 2005; Friedl et al., 2005; Padeshki et al., 2010; Chomel et al., 2016; Ehrensperger et al., 2018; Borgschulte et al., 2022).

*Francisella tularensis* isolation has been rarely reported from domestic animals and pets (O’Toole et al., 2008; Pennisi et al., 2013; Mani et al., 2016; Kwit et al., 2020). Direct human infections from these animals have most frequently involved cats and dogs, with the majority of cases reported in North America (Capellan and Fong, 1993; Liles and Burger, 1993; Arav-Boger, 2000; Mani et al., 2016; Yaglom et al., 2017; Kwit et al., 2019). These animals transmit tularemia to their owners through bites or scratches (especially when their owner tries to remove a small rodent from their mouth), but also probably by licking them in case of skin lesions. Numerous human infections have been reported in the Midwestern United States following bites or scratches from either feral or domestic cats (Mani et al., 2016). High tularemia seroprevalences have been reported in dogs and cats living in highly endemic rural areas, e.g., 14.2% in dogs, and 3.7% in cats in areas near public parks in Canada (Leighton et al., 2001). Cats and dogs are likely infected after preying on and consuming infected rabbits, hares, or rodents (Capellan and Fong, 1993; Liles and Burger, 1993; Mani et al., 2016; Kwit et al., 2019). When infected with *F. tularensis*, these animals may remain asymptomatic or develop mild to severe symptoms and even die from tularemia. Exotic pets have become more common in households and have carried new zoonotic risks for their owners (Chomel et al., 2007). Sometimes wild-caught animals potentially carrying or infected with *F. tularensis* have been sold as pets. For instance, prairie dogs taken from their natural habitats in the United States and subsequently sold as pets developed tularemia while in captivity (Centers for Disease Control and Prevention (CDC), 2002), leading to reported cases of the disease in humans (Avashia et al., 2004; Petersen et al., 2004).

Direct human contamination with *F. tularensis* from wild animals most often occurs in specific situations. Protective measures such as wearing gloves, glasses, and a mask when manipulating wildlife animals (especially game) or their carcasses should be considered. Although breeding and caring for pets is a less common source of human infection, the same protective measures should be considered by persons in contact with sick or dead pets at least until the etiological diagnosis has been established. Pet owners should be aware of the zoonotic risks associated with their animals. Veterinarians and their staff must take personnel protective precautions when manipulating sick or dead animals, especially cats and dogs. Tularemia should be considered following bites or scratches from domestic or wild animals, especially when individuals develop an infection in the affected skin area along with satellite lymphadenopathy. Amoxicillin-clavulanate, an antibiotic commonly used for prophylaxis against pasteurellosis and other bacterial infections, is ineffective against *F. tularensis*, whereas ciprofloxacin or doxycycline are effective options for tularemia prophylaxis (Ellis and Ellis, 2014).

## 6 Arthropod-borne tularemia: risk factors and prophylaxis

Many outdoor occupations and leisure activities potentially expose people to *F. tularensis*-contaminated arthropod vectors. Ixodidae ticks are the primary vectors of tularemia, with different tick species being implicated depending on the geographic region (Zellner and Huntley, 2019). Individuals in occupations that may frequently expose them to tick bites include farmers, forestry workers, game wardens, park rangers, landscapers, and military personnel. Leisure activities in areas where wildlife and ticks proliferate (including forests and meadows) expose people to tick-borne tularemia (Petersen et al., 2009). At risk activities include walking, cycling, and camping in a tick-infested forest. *F. tularensis*-infected ticks can also be carried and brought into homes by pets. The proportion of human cases of tularemia attributed to tick bites varies by geographic region, influenced by differences in the involved tick species, tick population density and the prevalence of *F. tularensis* infection in these arthropods. For instance, tick-borne tularemia is common in certain regions of the United States (Zellner and Huntley, 2019; Bishop et al., 2023), while it remains relatively rare in most European countries (Hestvik et al., 2014; Darmon-Curti et al., 2020).

Protective measures advocated for Lyme disease are also suitable for tickborne tularemia (Lantos et al., 2021). These two diseases often occur in the same geographical regions (Richard and Oppliger, 2015). People should wear long clothing that covers arms and legs when walking in grassy or wooded areas and use tick repellents. Recommended products approved for human use include N,N-Diethyl-meta-toluamide (DEET) and 3-(N-n-butyl-N-acetyl)-amino-propionic acid ethyl ester (IR3535) (Lantos et al., 2021). Other effective products include the para-methane-3,8-diol (PMD), permethrin, picaridin (KBR 3023), 2-undecanone (IBI-246), and oil of lemon eucalyptus (OLE) (Lantos et al., 2021). All these repellents have potentially severe side effects (notably neurological and skin toxicity) and should be used according to manufacturers’ recommendations. Not exceeding the maximum number of daily applications is critical especially in children and pregnant women. People should carefully examine their skin once they have returned home, especially on the scalp, behind the ears, in the armpits, between the legs, and behind the knees. Nymphs (1–3 mm size) are more likely to bite humans than adult ticks. Prompt removal of attached ticks, without squeezing or damaging them (e.g., using chemical products), must be done using a tick remover tool or a fine-tipped tweezer. People must be informed of the need to consult a doctor if they develop a fever, a skin lesion at the site of the tick bite, or regional lymphadenopathy. The risk of developing tularemia after a tick bite is challenging to assess. However, it is generally considered low due to the low prevalence of *F. tularensis* infection in ticks across most endemic regions, as well as the requirement for prolonged attachment (24 h or more) of the tick to its host for pathogen transmission. Therefore, in individuals who have been bitten by a tick, systematic identification of any collected tick, diagnostic testing, and antibiotic prophylaxis are not routinely recommended. Nonetheless, it is important to evaluate this risk on a case-by-case basis in each endemic region. For Lyme disease, where the likelihood of developing this borreliosis after a tick bite is considered significant, a single dose of doxycycline has been shown to be an effective prophylactic measure (Zhou et al., 2021).

Mosquito-borne tularemia is mainly encountered in specific regions of Sweden and Finland. Occupations and leisure activities near specific aquatic areas are particularly at risk (Desvars et al., 2015; Plymoth et al., 2024). Unlike ticks, a large proportion of the mosquito population living in these areas are likely infected with *F. tularensis* [see 3.3.2 and Thelaus et al. (2014)]. People living or staying in these tularemia endemic areas are advised to take prophylactic measures recommended for mosquito-borne diseases (Onen et al., 2023). The simplest measure is to avoid mosquito bites. Wearing significant clothing, using mosquito nets, and using repellents are classic and effective measures to avoid mosquito bites. Monitoring water points where mosquito larvae develop is also important. Mosquito repellents are the same products as those described above for ticks (Grison et al., 2020). DEET and IR3535 are also most often used. Picaridin is also very effective. The restrictions relating to the potential toxic effects of these products previously mentioned for ticks apply here. Clothing treated with these substances have a long-lasting efficacy. Since mosquitoes have developed resistance to some synthetic insecticides, notably DEET, bio-sourced repellents such as IR3535, permethrin, and PMD are now preferred (Grison et al., 2020).

## 7 Food-borne tularemia: risk factors and prophylaxis

Butchers, slaughterhouse workers, and renderers are occupations considered at risk of tularemia due to frequent exposure to animals, animal carcasses, and derived raw food products. Tularemia seroprevalences are usually higher in these professionals compared to the general population (Mattatia et al., 2024). Food products derived from game are more likely to be contaminated with *F. tularensis* than those from farm animals. However, there is not enough data to firmly establish what the usual modes of infection are among these professionals.

Food-borne tularemia cases have occurred after eating uncooked or undercooked food prepared from infected animals, usually game meat or other derived products (Djordjevic-Spasic et al., 2011; Maurin et al., 2011), or through the consumption of fruits such as apples (Cerný, 2001; Day et al., 2008) and grapes (Burckhardt et al., 2018). *F. tularensis* infections have been reported in people in contact with fruits and vegetable in processing plants (Levchenko, 1955; Cerný et al., 1986; Cerný, 1991). To our knowledge, food-borne tularemia linked to food products sold in stores has never been reported in the literature. To avoid food-borne tularemia, sick-looking game (often easier to capture) should not be hunted and consumed, game food should be handled with strict hygienic precautions, contact of game raw food with other common food products should be avoided, and such food should be cooked sufficiently and then stored at 4°C or below. For example, to guarantee food safety, it is recommended to cook meat at a core cooking temperature of 60–70°C (World Health Organization, 2024). *F. tularensis* is highly resistant to freezing and likely survives for long periods in frozen food specimens. Food (fruit and vegetables) picked up from the ground must be washed properly before consumption, especially if not cooked.

Tularemia cases related to the consumption *F. tularensis* contaminated water may be sporadic such as in Norway (Larssen et al., 2011) or occur as outbreaks such as in Turkey (Karadenizli et al., 2005; Erdem et al., 2014; Aktas et al., 2015; Kilic et al., 2015). These contaminations are usually linked to the consumption of non-chlorinated water contaminated by *F. tularensis*-infected small rodents. These include drinking water from springs, wells, fountains or water tanks. The large-scale epidemics observed in certain geographical areas, notably in Turkey, are linked to the population’s difficulties in accessing potable water, particularly due to defective water networks (Karadenizli et al., 2005; Erdem et al., 2014; Aktas et al., 2015; Kilic et al., 2015). Tularemia cases linked to the consumption of *F. tularensis*-contaminated water can be prevented by alerting the population living in tularemia endemic areas not to consume non-potable water, particularly from wells or springs. The prevention of tularemia epidemics requires the population’s access to sufficient drinking water and the installation or repair of water networks when necessary.

## 8 Tularemia of environmental sources: risk factors and prophylaxis

Land-based sources of *F. tularensis* can be responsible for acute, subacute, or even chronic respiratory infections or other clinical forms of tularemia. People can be contaminated for example through gardening work (mowing the lawn, clearing brush), handling fodder (notably hay), handling dead plants or compost, and cleaning a cellar or other enclosed spaces infested with small rodents (Dahlstrand et al., 1971; Syrjälä et al., 1985; McCarthy and Murphy, 1990; Feldman et al., 2001, 2003; Rossow et al., 2014). Severe acute respiratory infections were reported in landscapers during lawn mowing or brush cutting activities on Martha’s vineyard (McCarthy and Murphy, 1990; Feldman et al., 2001, 2003).

Waterborne infections caused by *F. tularensis* can occur following contact with surface water contaminated with this bacterium under a variety of conditions (Hennebique et al., 2019). Specifically, these infections have been reported following inhalation of water during drowning accidents, or from swimming or other recreational water activities such as canyoneering in stagnant and polluted waters. Fishing activities targeting species such as crayfish, pike, and walleye have also been associated with these infections. Affected patients have presented with a range of clinical manifestations, including pneumonic, ulceroglandular, and oropharyngeal forms of tularemia, and otitis media (Hennebique et al., 2019).

In tularemia endemic areas, the prevention of human infections contracted from the environment is challenging because the potential sources are difficult to identify (Dahlstrand et al., 1971; Syrjälä et al., 1985; McCarthy and Murphy, 1990; Feldman et al., 2001, 2003; Rossow et al., 2014; Hennebique et al., 2019). Respiratory infections are the most to be feared because they are potentially associated with severe infections for type A (Feldman et al., 2001) or chronic and debilitating infections for type B (Väyrynen et al., 2017; Martinet et al., 2021; Widerström et al., 2024). Individuals who may be exposed to aerosols contaminated with *F. tularensis* should wear a protective mask. In the high-risk scenarios discussed above, it is essential to avoid skin lesions by using appropriate gloves when handling soil or plants, and any damage that does occur should be properly disinfected and monitored. To prevent waterborne infections, it is advisable to avoid swimming or coming into contact with dirty, stagnant water where small aquatic rodents are known to proliferate. The risk of tularemia after exposure to the environmental reservoir of *F. tularensis* is little known by health professionals and even less by the general population. Specific information regarding this risk deserves to be disseminated at least to the medical community.

## 9 Laboratory-acquired infections: risk factors and prophylaxis

Laboratory personnel face a potential risk of contracting tularemia when handling *F. tularensis* cultures (Overholt et al., 1961; Pike et al., 1965; Morse and Henkel, 2018). Edward Francis who greatly contributed to the knowledge on *F. tularensis*, developed laboratory-acquired tularemia (Nelson and Sjöstedt, 2024). Laboratory infections with *F. tularensis* usually occur through skin inoculation or the inhalation of an infected aerosol (Overholt et al., 1961). Eye splashes or oral inoculation are other potential modes of infection.

*F. tularensis* subsp. *tularensis* is a class 3 biohazard agent and cultures of this pathogen must be handled in a biosafety level 3 (BSL-3) laboratory. In many countries, *F. tularensis* subsp. *holarctica* is a class 2 biohazard agent which can be handled in a BSL-2 laboratory. In the USA, all *F. tularensis* subspecies are considered a BSL-3 Tier 1 select agents, except for attenuated *F. tularensis* strains and opportunistic *F. novicida* (also known as *F. tularensis* subsp. *novicida*). Working with viable select agent strains requires compliance in many areas with the Federal Select Agent Program and the CDC in the USA.

In both cases, *F. tularensis* cultures should be performed under a biological safety hood, with appropriate personnel protective equipment including wearing gloves and a protective mask (Meechan and Potts, 2020). Manipulations potentially producing aerosols or droplets and centrifugation of infectious material must be done cautiously under a biological safety hood.

In research or reference laboratories, routine handling of *F. tularensis* cultures must be performed by a trained laboratory personnel, using specific procedures. This personnel must have regular medical surveillance and any potential exposure to *F. tularensis* must be reported immediately to decide whether antibiotic prophylaxis and clinical follow-up is necessary. In our experience, antibiotic prophylaxis should be restricted to cases of proven exposure to *F. tularensis* (e.g., inhalation of an aerosol, skin injury with contaminated needle or scalpel) whereas medical and serological surveillance is sufficient in most cases.

Vaccination with virulence-attenuated *F. tularensis* strains (such as the Live Vaccine Strain) is no longer approved for human use in most countries. In recent years, advancements such as the establishment of Biosafety Level 3 (BSL-3) facilities, improved safety cabinets, and stricter biosecurity protocols have significantly reduced the incidence of laboratory-acquired infections, including tularemia (Wurtz et al., 2016).

In clinical microbiology laboratories, laboratory staff should be alerted when a patient has suspected tularemia. Indeed, although rare, exposure to *F. tularensis* can occur during the handling of clinical samples from this patient without specific precautions (Morse and Henkel, 2018).

## 10 Health care personnels: risk factors and prophylaxis

Health professionals caring for tularemia patients might be exposed to *F. tularensis* contamination though the skin, conjunctiva, or the respiratory routes at the time of patient examination or during more invasive procedures (e.g., bronchoscopy or surgery). *F. tularensis* has been isolated from many clinical samples, including blood, skin eschars, throat or conjunctival swabs, lymph node or other organ specimens, cerebrospinal fluid, and pleural fluid. However, no cases of *F. tularensis* infection have been reported so far in healthcare providers in contact with a tularemia patient (Nelson et al., 2020). In fact, human-to-human transmission of *F. tularensis* in considered unlikely and has occurred only in two specific situations, including in patients transplanted inadvertently with organs taken from a person who died of tularemia (Nelson et al., 2019) and in a medical examiner during autopsy of a patient who died of tularemia (Weilbacher and Moss, 1938).

For routine caring of tularemia patients, health-care providers are only advised to use standard precautions (Nelson et al., 2020). Placement of patient with pneumonic tularemia in an airborne isolation room is not considered necessary. The usefulness of protecting skin ulcers with a dressing can be debated because of the occasional isolation of *F. tularensis* from these lesions. During invasive procedures, such as operations, autopsies and bronchoscopies, it seems prudent to advise healthcare professionals to use protective equipment such as gloves, glasses, specific suits and, in case of exposure to aerosols, a certified and protective respiratory mask. Accidental skin inoculation or aerosol exposure may prompt consideration of antibiotic prophylaxis.

## 11 Post-exposure antibiotic prophylaxis

Antibiotic prophylaxis is administered quickly to persons with a high likelihood of *F. tularensis* exposure to prevent symptoms onset although infection is not proven. If the risk of exposure to *F. tularensis* persists for several days (e.g., dispersion of an aerosol over a population) some people will be infected while already taking antibiotic prophylaxis. These two situations are difficult to differentiate in real life but have been evaluated in animal models.

### 11.1 Data in animal models

#### 11.1.1 Methodology

Antibiotic prophylaxis against tularemia has been primarily evaluated in mouse and non-human primate (NHP) models infected with the highly virulent type A strain Schu S4. The type B live vaccine strain (LVS) has also been used in mice because it is virulent in these animals. Animals were infected through the oral (po), respiratory (intranasal (in) or aerosol), subcutaneous (sc), intraperitoneal (ip), or intravenous (iv) routes. The effectiveness of antibiotics administered to animals before or after infection with *F. tularensis* was evaluated based on the occurrence of symptoms, relapse and death rates, and bacterial loads in different organs. Experimental conditions ensured 100% mortality in infected and untreated control animals.

#### 11.1.2 Mouse models

##### 11.1.2.1 Pre-challenge antibiotic prophylaxis

In BALB/c mice challenged with the LVS strain (10^2^ CFU, in or 10^3^ CFU, iv), a single 1 mg dose (iv or in) of ciprofloxacin given 1, 2, 3, or 7 days before infection only allowed 0–12% survival rates (Di Ninno et al., 1993) (Table 1). In Porton outbred mice infected with a low dose (10–10^3^ CFU, ip) of Schu S4, ciprofloxacin and doxycycline (20 or 40 mg/Kg bid, sc, for 7 days) given 48 h before infection rescued 100% and 13.3–73.3% of animals, respectively (Russell et al., 1998). Both antibiotics were poorly active in animals infected with a high Schu S4 inoculum (10^5^–10^7^ CFU, ip). Overall, the pre-exposure prophylaxis was more effective for low *F. tularensis* infecting doses and when prolonged for 1 week after infection. Additionally, ciprofloxacin was found to be more effective than doxycycline (Di Ninno et al., 1993; Russell et al., 1998).

**Table 1:** Antibiotic prophylaxis efficacy against *Francisella tularensis* infection in mice.

Drugs	Dosage, route^$^	Duration	Time before or post-infection	Survival rate in animals depending on treatment time	Statistical significance versus untreated controls (100% death rates) or between antibiotics when specified
Barnes et al. (2021), Balb/c mice, Schu S4, aerosol, ~300 CFU					
Finafloxacin	23.1 mg/kg tid, po	3 days	1 dpi^$^	100% at 35 dpi	*p* < 0.0001
		7 days	1 dpi	100% at 35 dpi	*P* < 0.0001
		3 days	3 dpi	0% at 35 dpi	NS, but delayed death
		7 days	3 dpi	50% at 35 dpi	More active than ciprofloxacin for 7 days (*p* < 0.01%)
Ciprofloxacin	30 mg/kg bid, ip	3 days	1 dpi	100% at 35 dpi	*P* < 0.0001
		7 days	1 dpi	100% at 35 dpi	*P* < 0.0001
		3 days	3 dpi	0% at 35 dpi	NS
		7 days	3 dpi	10% at 35 dpi	NS
Grossman et al. (2017)^£^, BALB/c mice, Schu S4, aerosol, ~1,000 CFU					
TP-271 fluorocycline (MIC = 0.03 μg/mL)	3 mg/kg od, ip	21 days	1 dpi	80% at 21 dpi and 50.4% at 37 dpi	*P* < 0.001 for both
			3 dpi	89% at 21 dpi and 37 dpi	*p* < 0.0001 for both; more active than doxycycline at 37 dpi (*p* = 0.01)
	6 mg/kg od, ip	21 days	1 dpi	100% at 21 dpi and 37 dpi	*p* < 0.0001 for both
			3 dpi	100% at 21 dpi and 89% at 37 dpi	*p* < 0.0001 for both
	12 mg/kg od, ip	21 days	1 dpi	100% at 21 dpi and 80% at 37 dpi	*p* < 0.0001 for both
			3 dpi	100% at 21 dpi and 37 dpi	*p* < 0.0001 for both; more active than doxycycline at 37 dpi (*p* = 0.007)
	18 mg/kg od, ip	21 days	1 dpi	100% at 21 dpi and 37 dpi	*p* < 0.0001 for both
			3 dpi	100% at 21 dpi and 37 dpi	*p* < 0.0001 for both; more active than doxycycline at 37 dpi (p = 0.01)
doxycycline	40 mg/kg bid, ip	21 days	1 dpi	84% at 21 dpi and 73.9% at 37 dpi	*p* < 0.0001 for both
			3 dpi	100% at 21 dpi and 22% at 37 dpi	*p* < 0.0001 for both
Hamblin et al. (2014), BALB/c mice, LVS, intranasal, 6 × 10^4^ CFU					
Ciprofloxacin	50 mg/Kg, po	single dose	3 or 4 dpi	100% at 21 dpi	*p* < 0.05
Hamblin et al. (2014), BALB/c mice, Schu S4, aerosol, 10 CFU					
Ciprofloxacin	50 mg/Kg, po	Single dose	1 dpi	0% at 28 dpi	NS
	50 mg/Kg bid, po	3 days	1 dpi	0% at 28 dpi, delayed death	NS
	50 mg/Kg bid, po	5 days	1 dpi	18% at 28 dpi, delayed death	NS
Crane et al. (2012), C57 Bl/6 mice, Schu S4, intranasal, 50 CFU					
Levofloxacin	40 mg/kg, ip	14 days	1, 2, or 3 dpi	100% at 30 dpi	*p* < 0.05 for both
	5 mg/kg, ip	14 days	1, 2, or 3 dpi	100, 100, and 60% at 30 dpi	*p* < 0.05 for all
Rotem et al. (2012), BALB/c mice, LVS, intranasal, 10^5^ CFU					
Ciprofloxacin	50 mg/Kg bid, ip	7 days	1, 2, or 3 dpi	100, 100, 100%	*p* < 0.05
Doxycycline	40 mg/Kg bid, ip	14 days	1, 2, or 3 dpi	100, 100, 100%	*p* < 0.05
Rotem et al. (2012), BALB/c mice, Schu S4, intranasal, 10^2^ CFU					
Ciprofloxacin	50 mg/Kg bid, ip	7 days	1, 2, or 3 dpi	100, 100, 70%	*p* < 0.05
Ciprofloxacin	50 mg/Kg bid, ip	10 days	3dpi	100%, no relapse	*p* < 0.05
Doxycycline	40 mg/Kg bid, ip	14 days	1, 2, or 3 dpi	90, 30, 0%	*p* < 0.05 for 1 and 2 dpi; NS for 3 dpi
Doxycycline	40 mg/Kg bid, ip	21 days	3 dpi	10%	NS
Sutherland et al. (2012), BALB/cJ mice, Schu S4, intranasal, 50 CFU					
Gentamicin	5 mg/Kg/day, ip	10 days	6hpi, 12hpi, 1dpi, 2dpi	0% at 25 dpi with death time like untreated control	NS
	10 mg/Kg/day, ip	10 days	6hpi, 12hpi, 1dpi, 2dpi	0% for 12 hpi, 1 dpi, and 2 dpi; and 20% for 6 hpi, at 25 dpi	
	20 mg/Kg/day, ip	10 days	12hpi, 1dpi, 2dpi	100% for 12 hpi and 1 dpi only	
	40 mg/Kg/day, ip	10 days	6hpi, 12hpi, 1dpi, 2dpi	100%	
Peterson et al. (2010), BALB/c mice, Schu S4, intranasal, 1.7 × 10^2^ CFU					
Levofloxacin	0.1 mg/kg/day, ip	13 days	1 dpi	54% at 26 dpi	NS
	0.5–10 mg/kg/day, ip	13 days	1 dpi	100% at 26 dpi (except 90% at 1 mg/Kg/day)	*p* < 0.05
	40 mg/kg/day, ip	13 days	1, 2, 3, 4 or 5 dpi	100, 100, 100, 80, and 0% at 70 dpi	*p* < 0.05 for 1, 2, 3, and 4 dpi; NS for 5 dpi
Klimpel et al. (2008), BALB/c mice, Schu S4, intranasal, ~100 CFU					
Levofloxacin	50, 25, 12.5, and 6.25 mg/kg/day, ip	13 days	1 dpi	100%	*p* < 0.0001
	40 mg/kg/day, ip	13 days	1, 2, 3, 4, or 5 dpi	100, 100, 100, 80, and 0% at 40 dpi	*p* < 0.0001 for 1, 2, 3, and 4 dpi; NS for 5 dpi
Steward et al. (2006), BALB/c mice, Schu S4, aerosol, 1.5×10^4^ CFU					
Ciprofloxacin	100 mg/Kg bid, po	14 days	6 hpi^$^, or 1 or 2 dpi	0% at 42 dpi	
Moxifloxacin	100 mg/Kg bid, po	14 days	6 hpi, or 1 or 2 dpi	53, 12, and 35% at 42 dpi	More active than ciprofloxacin (*p* < 0.01)
Gatifloxacin	100 mg/Kg bid, po	14 days	6 hpi, or 1 or 2 dpi	53, 41, and 65% at 42 dpi	More active than ciprofloxacin (*p* < 0.01) except for 1 dpi
Piercy et al. (2005), BALB/c mice, Schu S4, subcutaneous, 10^6^ CFU					
Ciprofloxacin	100 mg/kg tid, po	14 days	6 hpi, or 1 or 2 dpi	94, 67, and 0% at 42 dpi	*P* < 0.001 for 6 hpi and 1 dpi; NS for 2 dpi
Moxifloxacin	100 mg/kg tid, po	14 days	6 hpi, or 1 or 2 dpi	100, 100, and 62% at 42 dpi	*p* < 0.001 for 6 hph, and 1 and 2 dpi; more active than ciprofloxacin for 1 and 2 dpi (*p* < 0.05)
Gatifloxacin	100 mg/kg tid, po	14 days	6 hpi, or 1 or 2 dpi	100, 96, and 84% at 42 dpi	*p* < 0.001 for 6 hpi, and 1 and 2 dpi; more active than ciprofloxacin for 1 and 2 dpi (*p* < 0.05)
Russell et al. (1998) §, Porton outbred mice, Schu S4, intraperitoneal, ~10–10^3^ CFU					
Doxycycline subcutaneous	40 mg/Kg bid, sc	7 days	48 h before challenge	73.3% at 24 dpi	
	20 mg/Kg bid, sc	7 days	48 h before challenge	13.3% at 24 dpi	
	40 mg/Kg bid, sc	5 days	1 dpi	93.3% at 24 dpi	
	20 mg/Kg bid, sc	5 days	1 dpi	60% at 24 dpi	
Ciprofloxacin subcutaneous	40 mg/Kg bid, sc	7 days	48 h before challenge	100% at 24 dpi	
	20 mg/Kg bid, sc	7 days	48 h before challenge	100% at 24 dpi	
	40 mg/Kg bid, sc	5 days	1 dpi	100% at 24 dpi	
	20 mg/Kg bid, sc	5 days	1 dpi	93.3% at 24 dpi	
Russell et al. (1998) §, Porton outbred mice, Schu S4, intraperitoneal, 10^5^–10^7^ CFU					
Doxycycline subcutaneous	40 mg/Kg bid, sc	7 days	48 h before challenge	93.3 at 24 dpi	
	20 mg/Kg bid, sc	7 days	48 h before challenge	53.3% at 24 dpi	
	40 mg/Kg bid, sc	5 days	1 dpi	60% at 24 dpi	
	20 mg/Kg bid, sc	5 days	1 dpi	33.3% at 24 dpi	
	40 mg/Kg bid, sc	10 days	1 dpi	100% at 24 dpi	
Ciprofloxacin subcutaneous	40 mg/Kg bid, sc	7 days	48 h before challenge	73.3% at 24 dpi	
	20 mg/Kg bid, sc	7 days	48 h before challenge	53.3% at 24 dpi	
	40 mg/Kg bid, sc	5 days	1 dpi	46.6% at 24 dpi	
	20 mg/Kg bid, sc	5 days	1 dpi	73.3% at 24 dpi	
	40 mg/Kg bid, sc	10 days	1 dpi	100% at 24 dpi	
Di Ninno et al. (1993), BALB/c mice, LVS, 10^2^ CFU intranasal or 10^3^ CFU intravenous					
Ciprofloxacin	1 mg, iv	Single dose	1, 2, 3, and 7 days before infection	8, 0, 12, 0%	NS
Ciprofloxacin	1 mg, in	Single dose	1, 2, 3, and 7 days before infection	0, 12, 0, 12%	NS
Liposomal ciprofloxacin	1 mg, iv	Single dose	1, 2, 3, and 7 days before infection	92, 100, 25, 0%	More active than ciprofloxacin for 1 and 2 days before infection (*p* < 0.005)
Liposomal ciprofloxacin	1 mg, in	Single dose	1, 2, 3, and 7 days before infection	92, 83, 100, 63%	More active than ciprofloxacin for 1, 2 and 3 days before infection (*p* < 0.005), and for 7 days before infection (*p* < 0.01)
Ciprofloxacin	1 mg, iv	Single dose	1, 2, 3, or 7 dpi	0, 25, 0, 12%	
Ciprofloxacin	1 mg, in	Single dose	1, 2, 3, or 7 dpi	50, 0, 25, 0%	
Liposomal ciprofloxacin	1 mg, iv	Single dose	1, 2, 3, or 7 dpi	75, 88, 0, 0%	More active than ciprofloxacin for 1 and 2 dpi (*p* < 0.005)
Liposomal ciprofloxacin	1 mg, in	Single dose	1, 2, 3, or 7 dpi	83, 100, 63, 50%	More active than ciprofloxacin for 2 dpi (*p* < 0.005) and 1 and 3 dpi (*p* < 0.01)

$: hpi: hours post-infection; dpi: days post-infection; od: once a day; bid: twice a day; tid: three times a day; po: orally; ip: intraperitoneally; in: intranasally; sc: subcutaneously; iv: intravenously. £: the original manuscript indicates the percentages of surviving animals at end of treatment (21 dpi) and among them the percentage of animals that were still alive at 37 dpi (16 days after treatment cessation). §: in this study, antibiotic efficacy was evaluated with different challenge doses (~10 to 10^7^ CFU) of Schu S4. Here, we have combined data obtained in animals exposed to low doses (10–10^3^ CFU) or high doses (10^5^–10^7^ CFU) of Schu S4 strain, corresponding to 15 animals for each prophylactic treatment group. The LD50 (i.e., lethal dose 50%, corresponding to the dose of the tested compound at which half the tested animals are killed) was determined to be close to 1 CFU.

##### 11.1.2.2 Post-challenge antibiotic prophylaxis in LVS-infected BALB/c mice

In mice infected with 10^2^ CFU in or 10^3^ CFU iv, a single 1 mg dose of ciprofloxacin given at 1, 2, 3 or 7 days post-infection (dpi) rescued 0–50% and 0–25% animals, when given in or iv, respectively (Di Ninno et al., 1993) (Table 1). In another study, a single 50 mg/Kg dose of ciprofloxacin given up to 4 dpi was fully effective in mice infected with 6 × 10^4^ CFU intranasal Hamblin et al. (2014). All mice infected with 10^5^ CFU in were rescued by ciprofloxacin (50 mg/Kg, bid, ip) for 7 days or doxycycline (40 mg/Kg, bid, ip) for 14 days, when the antibiotics were administrated at 1, 2, or 3 dpi (Rotem et al., 2012). Interestingly, liposomal ciprofloxacin was more effective than free ciprofloxacin (Di Ninno et al., 1993). Overall, in LVS-infected mice, ciprofloxacin and doxycycline were effective prophylaxis, although longer administration was required for doxycycline.

##### 11.1.2.3 Post-challenge antibiotic prophylaxis in Schu S4-infected mice

In Schu S4-infected BALB/c mice (50 CFU, in), gentamicin was fully effective when given within 48 h after infection, at high dosage (40 mg/Kg/day) for 10 days (Sutherland et al., 2012) (Table 1). Doxycycline rescued almost all BALB/c mice infected with a low dose (10^2^ CFU in, or 10^3^ CFU aerosol) of Schu S4 only when given at high dosage (80 mg/Kg/day), for 2–3 weeks, and within 24 h post-challenge (Rotem et al., 2012; Grossman et al., 2017). In Porton outbred mice infected intraperitoneally with a low (10–10^3^ CFU) or high (10^5^–10^7^ CFU) Schu S4 inoculum, doxycycline rescued all animals when given 24 h post-challenge at 80 mg/Kg/day for 10 days (Russell et al., 1998). Doxycycline was much less effective when given at lower dosages and duration (40 mg/Kg/day for 5 days) (Russell et al., 1998) or more than 24 h post-challenge (Rotem et al., 2012; Grossman et al., 2017).

The fluoroquinolones were usually more effective than doxycycline in Schu S4-infected mice (Russell et al., 1998; Rotem et al., 2012). Ciprofloxacin was fully effective in BALB/c mice infected with a low (100–1,000 CFU) Schu S4 dose when given at high (60–100 mg/Kg/day) concentration for 7 days and within 48 post-challenge (Rotem et al., 2012; Barnes et al., 2021). Full efficacy was still observed when this antibiotic was given 3 days post-challenge at the same dosage but for 10 days (Rotem et al., 2012). In Porton outbred mice, ciprofloxacin was fully effective in animals challenged with 10–10^7^ CFU of Schu S4 only when given within 24 h post-challenge, at 80 mg/Kg/day for 10 days (Russell et al., 1998). In other studies, however, ciprofloxacin was much less effective when given 24-48 h after BALB/c mice were infected with a high (1.5 × 10^4^ CFU aerosol, or 10^6^ CFU sc) Schu S4 inoculum, despite being administrated at high dosage (200–300 mg/Kg/day) for 14 days (Piercy et al., 2005; Steward et al., 2006). Liposomal ciprofloxacin was more effective than free ciprofloxacin (Hamblin et al., 2014).

Levofloxacin (6.25–50 mg/kg/day, ip, for 13 days) rescued all BALB/c mice infected with 100 CFU, in, when given at 1 dpi (Klimpel et al., 2008). At 40 mg/Kg/day, this antibiotic remained effective when given at 1–4 dpi. Lower dosages of levofloxacin (0.5 to 10 mg/Kg/ day, ip, for 13 days) given 24 h after a 100 CFU in challenge also rescued all animals (Peterson et al., 2010). In C57Bl/6 mice challenged with 50 CFU in, levofloxacin rescued all animals when given for 14 days at 40 mg/Kg/day, 1–3 dpi, but only 60% animals at 5 mg/Kg/day given at 3 dpi (Crane et al., 2012). Among new fluoroquinolones, gatifloxacin and moxifloxacin were significantly more effective than ciprofloxacin (Piercy et al., 2005; Steward et al., 2006), but not finafloxacin (Barnes et al., 2021). The fluorocycline TP-271 rescued all mice infected with a 10^3^ CFU aerosol when given at 1 dpi or 3 dpi (Grossman et al., 2017). The superiority of levofloxacin, gatifloxacin, and moxifloxacin over ciprofloxacin observed in mouse models deserves further evaluation.

Overall, the above data indicate that a fluoroquinolone was the best oral alternative for tularemia prophylaxis in Schu S4-infected mice provided it was administrated early (within 3 days) after challenge, at appropriate dosage, and for 10–14 days. It’s to be noted that in mouse models, experimental conditions were highly variable. *F. tularensis* infection was performed by different routes, i.e., aerosol, intranasal, or intraperitoneal. Antibiotics were also administrated by different routes, i.e., oral, intraperitoneal, subcutaneous, intranasal, or intravenous. The animals were infected either with the LVS or the Schu S4 strain of *F. tularensis*. The incubated bacterial load varied from 10 CFU up to 10^7^ CFU. Comparisons of results obtained in these different models is challenging. However, the route of *F. tularensis* infection has a significant impact on the results. Considering only mice infected with the Schu S4 strain and treated 1 day post-infection, results indicate that antibiotics were more effective when the animals were challenged by the subcutaneous or intraperitoneal routes, compared to the respiratory route (aerosol or intranasal). At the lowest bacterial inoculum (10 to 1,000 CFU) doxycycline and ciprofloxacin usually displayed similar activity whatever the route of infection (Russell et al., 1998; Rotem et al., 2012; Grossman et al., 2017; Barnes et al., 2021). However, in one study, only 18% of mice infected with a 10 CFU aerosol and treated with 50 mg/Kg bid of ciprofloxacin for 5 days survived (Hamblin et al., 2014). In contrast, all mice infected intraperitoneally with 10–1,000 CFU and treated with 40 mg/Kg bid of ciprofloxacin for 5 days survived (Russell et al., 1998). The difference was more pronounced for mice infected with a high *F. tularensis* inoculum. All mice infected with a 1.5 × 10^4^ CFU aerosol and treated with 100 mg/Kg bid for 14 days died (Steward et al., 2006). Mice infected subcutaneously with 10^6^ CFU and receiving the same antibiotic treatment had a 67% survival rate (Piercy et al., 2005). Mice infected intraperitoneally with 10^5^–10^7^ CFU and treated with 40 mg/Kg bid of ciprofloxacin for only 5 days had a 46.6% survival rate (Russell et al., 1998).

In NHPs models and human volunteers (see below), the primary goal was to test the antibiotic efficacy after infection with the most virulent Schu S4 strain via the respiratory route, which is considered a natural route of infection leading to the most severe infections. The primary goal was to determine which antibiotic prophylaxis or early therapy would be the most effective in an epidemic situation, particularly after the intentional release of *F. tularensis* aerosols. The question arises as to which mouse model is closest to this situation and therefore most predictive of the efficacy of antibiotic prophylaxis to prevent symptomatic infections and death after exposure to an aerosol of this pathogen. It would seem desirable to consider the following criteria as optimum: 1/ an infection by a highly virulent type A strain (e.g., the Schu S4 strain), by the respiratory route (aerosol or intranasal), using a low or high *F. tularensis* inoculum, capable of inducing severe symptoms and a high risk of death within a few days in the absence of treatment; 2/ an antibiotic administered within 24 to 48 h post-infection, for at least 1 week, orally or intravenously, and at a concentration allowing pharmacokinetics close to that in humans; and 3/ an efficacy objective including a survival rate close to 100% and the absence of the appearance of severe symptoms. These criteria can be found in many studies, at least for some of the animals tested (Steward et al., 2006; Klimpel et al., 2008; Peterson et al., 2010; Crane et al., 2012; Rotem et al., 2012; Sutherland et al., 2012; Grossman et al., 2017; Barnes et al., 2021).

#### 11.1.3 Non-human primate models

In Rhesus macaques (*Macaca mulatta*) infected with a 10^4^ CFU Schu S4 aerosol, tetracycline (200 mg intragastric every 24 h, 36 h, 48 h, or 72 h, for 13 days) rescued most animals (83.2–100%) when given at 1 dpi (Sawyer et al., 1966) (Table 2). However, symptoms developed in 90.9% animals treated every 24-48 h on antibiotic withdrawal, and in all animals treated every 48-72 h while under treatment. When tetracycline treatment (200 mg/day, 13 days) started at 2.5 dpi, only 66.6% animals survived.

**Table 2:** Antibiotic prophylaxis efficacy against *Francisella tularensis* infection in non-human primate models.

Drugs	Dosage^$^, route^$^	Duration	Time pi	Survival rate in treated *vs* untreated animals (p)
Jakielaszek et al. (2023), Cynomolgus macaque, Schu S4, aerosol, ~1,000 CFU				
Gepotidacin ^*^	22 mg/kg, then 3.5 h later 2 mg/kg tid, iv (total dose 72/mg/kg/day)	10 days	1 dpi	100% vs. 12.5% at 43 dpi (p < 0.001), fever on 2–3 dpi, resolved 7 dpi under treatment
Grossman et al. (2017), Cynomolgus macaque, Schu S4, aerosol, ~1,000 CFU				
TP-271 fluorocycline ^**^	1 mg/kg, od, iv	21 days	Within 6 h of fever (2–4 dpi)	100% at d21 and d37 vs. 0% (*p* = 0.0002)
	3 mg/kg, od, iv	21 days	Within 6 h of fever (2–4 dpi)	100% at d21 and d37 vs. 0% (p = 0.0002)
Nelson et al. (2010), Common marmoset, Schu S4, aerosol, ~300 CFU				
Levofloxacin	16.5 mg/Kg, bid, po	10 days	1 dpi	100% vs. 0% at 14 dpi
Baskerville et al. (1978), Grivet, Schu S4, intranasal, 5 × 10^4^ CFU				
Kanamycin	70 mg od, im	7 days	3 or 4 dpi	100% at 24 dpi
Baskerville et al. (1978), Grivet, Schu S4, intranasal, 5 × 10^4^ CFU				
Kanamycin	70 mg od, im	7 days	3 or 4 dpi	100% (3 dpi) and 75% (4 dpi) at 21 dpi vs. 0% at 5–7 dpi. Lymph node abscesses in two animals at 14–21 dpi requiring surgery.
Sawyer et al. (1966), Rhesus macaque, Schu S4, aerosol, 10.000 CFU				
Tetracycline	200 mg ig, dosage interval of 24 h, 36 h, 48 h, or 72 h	13 days	1 dpi	100, 83.3, 83.3, and 100% for 24 h, 36 h, 48 h, and 72 h dosage interval, respectively, vs. 0%; variable efficacy on symptoms (see text)
	200 mg per day ig	13 days	60 hpi	66.6% versus 0%
Sawyer et al. (1966), Rhesus macaque Schu S5, aerosol, 10.000 CFU				
Tetracycline	75 mg tid, ig	7 days or 3 days no fever	After 12 h of fever >40°C or when fever >41°C	100%, but all animals experienced relapses
Kanamycin	30 mg tid, ig			100%, no relapse
Gentamicin	3 mg tid, ig			100%, but 37.5% relapses

^*^Gepotidacin activity against 129\u00B0F. tularensis type A and type B strains: MIC range, 0.06–4 mg/L; MIC50, 0.25 mg/L; MIC90, 0.5 mg/L. ^**^TP-271 (fluorocycline) activity against 29\u00B0F. tularensis strains: MIC50, 0.25 mg/L; MIC90, 0.5 mg/L. $: hpi: hours post-infection; dpi: days post-infection; od: once a day; bid: twice a day; tid: three times a day; po: orally; iv: intravenously; im: intramuscularly; ig: intragastrically.

Rhesus macaques infected with a 10^4^ CFU Schu S5 aerosol, a streptomycin-resistant strain, were all rescued when treated within 3 dpi with tetracycline (225 mg daily), kanamycin (90 mg daily) or gentamicin (9 mg daily) for 7 days or until fever abated (Sawyer et al., 1966). However, relapse rates were 100% for tetracycline, 37.5% for gentamicin, and 0% for kanamycin.

In grivets (*Chlorocebus aethiops*) infected intranasally with 5 × 10^4^ CFU of Schu S4, kanamycin (70 mg per day, intramuscular, for 7 days) rescued all animals (Hambleton et al., 1978). In a similar study (Baskerville et al., 1978), kanamycin cured three animals treated at 3 dpi, whereas among four animals treated at 4 dpi, one died and two developed lymph node abscesses 2–3 weeks post-infection requiring surgical drainage and streptomycin for cure.

In common marmoset (*Callithrix jacchus*) infected with a 300 CFU aerosol of Schu S4, levofloxacin (33 mg/Kg/day orally for 10 days) administrated at 1 dpi allowed a 100% survival rate, no symptoms onset and absence of *F. tularensis* in organs collected from animals euthanized at 24 dpi (Nelson et al., 2010). This bacterial inoculum was sufficient to kill all untreated control animals within five dpi.

In Cynomolgus macaques (*Macaca fascicularis*), infected with a 1,000 CFU Schu S4 aerosol, gepotidacin (72 mg/Kg/day for 10 days), a new type II topoisomerase inhibitor, rescued all animals when given at 1 dpi (Jakielaszek et al., 2023). In a similar model, the fluorocycline TP-271 (1 or 3 mg/Kg/day for 21 days) rescued all animals when given at 2–4 dpi (Grossman et al., 2017).

Overall, in the above NHP models, tetracycline (200–225 mg per day) and the aminoglycosides gentamicin (9 mg daily) and kanamycin (70–90 mg daily) were fully effective when given within 3 dpi for at least 7 days (Sawyer et al., 1966). However, almost all animals treated with tetracycline developed symptoms suggesting this antibiotic only had an *in vivo* bacteriostatic activity (Sawyer et al., 1966). Symptoms onset were less frequently observed with gentamicin and kanamycin, suggesting higher *in vivo* efficacy (Sawyer et al., 1966; Baskerville et al., 1978; Hambleton et al., 1978). All NHPs treated with levofloxacin (33 mg/Kg/day for 10 days) survived without any symptoms and were cured from *F. tularensis* infection at 24 dpi, suggesting a strong *in vivo* bactericidal activity (Nelson et al., 2010). Since antibiotic doses used in NHPs are close to those administered in humans, NHP models might be more relevant than mouse models to predict antibiotic prophylaxis efficacy in humans. However, both models support the use of a fluoroquinolone as a first-line post-exposure prophylaxis of tularemia.

### 11.2 Human data

The antibiotic prophylaxis against tularemia has been evaluated in humans volunteers (McCrumb et al., 1957; McCrumb, 1961; Sawyer et al., 1966; Williams et al., 2019) (Table 3). Although this type of study is no longer carried out today for ethical reasons, the data obtained from these volunteers deserves to be mentioned. In most studies, people were infected with a 25,000 CFU Schu S4 aerosol leading to fever onset at approximately three days post-challenge (Williams et al., 2019).

**Table 3:** Antibiotic prophylaxis efficacy against *Francisella tularensis* infection in humans.

Drugs	Dosage, route	Duration	Time pi	Illness during treatment, n/tested (%)	Illness after treatment withdrawal, n/tested (%)
Sawyer et al. (1966), Schu S4, aerosol, 25,000 CFU					
Tetracycline	500 mg bid, NA	15 days	1 dpi	0/10 (0%)	2/10 (20%)
	500 mg bid, NA	28 days	1 dpi	0/8 (0%)	0/8 (0%)
	1 g bid, NA	14 days	1 dpi	0/8 (0%)	0/8 (0%)
	500 mg bid, every other day, NA	19 days	1 dpi	2/8 (25%)	8/8 (100%)
Sawyer et al. (1966), Schu S4 or Schu S5, aerosol, 25,000 CFU					
Tetracycline	4 g the first day then 500 mg qid, NA	10 days	Within 48 h of fever onset (occurring 2–7 dpi)	Relapses in 5/11 (45.5%)	
	4 g the first day then 500 mg qid, NA	15 days		Relapses in 0/20 (0%)	
	4 g the first day then 250 mg qid, NA	15 days		Relapses in 2/8 (25%)	
Williams et al. (2019), Schu S4, aerosol, 25,000 CFU					
Tetracycline	0.25 g qid, po	≥14 days	5 dpi	5/11 (45.5%)	6/11 (54.5%)
	0.5 g qid or 1 g bid, po	<14 days	5 dpi	2/8 (25%)	1/8 (12.5%)
	0.5 g qid or 1 g bid, po	≥14 days	5 dpi	0/44 (0%) better than 1 g/day ≥14 days (*p* = 0.02) and 2 g/day <14 days (*p* < 0.001)	14/55 (25.5%), better than 2 g/day <14 days (*p* < 0.05)

$: dpi: days post-infection; bid: twice a day; qid: four times a day; po: orally; im: intramuscularly; NA: route of antibiotic administration unspecified (likely orally).

Tetracycline was fully effective to prevent symptoms onset when given at 1 dpi at either 1 g per day for 28 days or 2 g per day for 14 days (Sawyer et al., 1966). At 1 g per day for 15 days, 20% of volunteers developed symptoms after antibiotic treatment withdrawal. At a lower dosage (1 g every two days for 19 days), 25% of volunteers developed symptoms under treatment and 100% after treatment withdrawal. When tetracycline was administrated 2–7 dpi, relapse rates were 0% at 2 g per day for 15 days, 25% at 1 g per day for 15 days, and 50% at 2 g per day for 10 days.

Williams et al. (2019) summarized data from studies conducted between 1958 and 1968 at Fort Detrick (Maryland, United States), the center of the USA biological weapons program until 1969. Tetracycline was given daily beginning 5 dpi, at a 1 g or 2 g dose, for a period of either less or more than 14 days. About half (45.5%) volunteers receiving 1 g per day of tetracycline relapsed after treatment withdrawal and required streptomycin for cure.

At 2 g daily of tetracycline, none (*p* < 0.001) of the patients relapsed when treated for more than 14 days, and 25% relapsed when treated for less than 14 days. Regardless of the treatment regimen employed, many patients experienced a recurrence of symptoms after stopping tetracycline. Only those who required further antibiotic treatment to achieve a cure were classified as true relapse cases by the authors. Nevertheless, the resurgence of symptoms following the discontinuation of antibiotic therapy in many patients strongly suggests reactivation of bacterial multiplication, underscoring the bacteriostatic effect of tetracycline on *F. tularensis*.

## 12 Discussion

**Figure 1:**
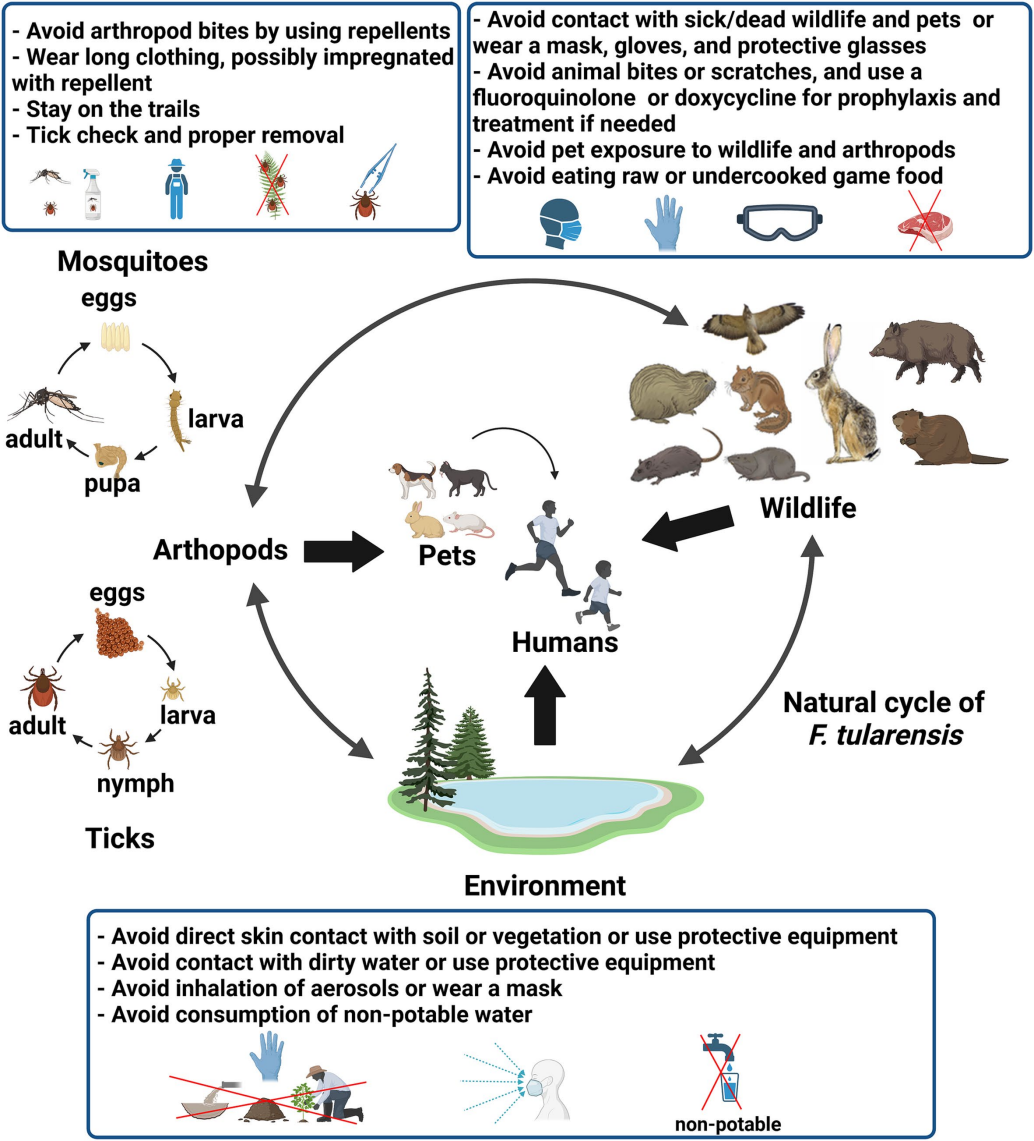
Figure showing the natural cycle of *F. tularensis*, the sources and modes of human infection by this bacterium, and the main prophylactic measures against tularemia. The figure was created using the Scientific Image and Illustration Software BioRender (https://www.biorender.com).

Tularemia is a rare and sporadic disease in most endemic countries with mosquito-borne outbreaks in Sweden and Finland, and water-borne outbreaks in Turkey as exceptions (Erdem et al., 2014; Desvars et al., 2015). *F. tularensis* has an extended reservoir including wild animals, arthropod vectors, and the environment (Hennebique et al., 2019; Telford and Goethert, 2020). Thus, the modes of human infection are varied, including contact with infected animals, scratches and bites from these animals, ingestion of contaminated water or food, inhalation of contaminated aerosols, contact with a contaminated environment, and arthropod bites (Sjöstedt, 2007; Erdem et al., 2014; Hennebique et al., 2019; Zellner and Huntley, 2019). The variable routes of infection (i.e., the skin, the airways and digestive tracts, and the conjunctiva) determined the clinical manifestations (Maurin and Gyuranecz, 2016; Wu et al., 2024). As a result, the prevention of human tularemia cases depends on the predominant modes of contamination in a specific geographic areas. In this review we have summarized the data relating to the known reservoirs of *F. tularensis*, the risk factors and modes of transmission of this pathogen to humans, and the existing or potential individual or collective prophylactic measures for tularemia (Figure 1).

Although research into the development of tularemia vaccines is very active (Conlan, 2011; Marohn and Barry, 2013; Jia and Horwitz, 2018), none are currently approved for human use. Vaccination with virulence-attenuated type B strains has limited effectiveness in preventing severe type A infections and their effectiveness decreases over time, requiring regular booster vaccinations (Conlan, 2011; Marohn and Barry, 2013; Jia and Horwitz, 2018). Antibiotic prophylaxis is considered effective when administrated early after proven or very likely exposure to *F. tularensis*, which might occur for example in the laboratory personnel handling *F. tularensis* cultures or in a biological threat context (Dennis et al., 2001; Bossi et al., 2004). Current recommendations are the early administration of a fluoroquinolone for 2 weeks (Dennis et al., 2001; Bossi et al., 2004). Doxycycline administered orally at 200 mg per day for 2 to 3 weeks is an alternative, but the above experimental and human data clearly indicate the superiority of fluoroquinolones to tetracyclines. The selection of one of these two classes of antibiotics remains difficult for either prophylaxis or therapeutic purposes and should also consider their contraindications and potential side effects (Maurin et al., 2023). Both tetracyclines and fluoroquinolones may trigger allergic reactions and gastrointestinal disturbances. Fluoroquinolones are associated with an increased risk of tendonitis and tendon ruptures. Tetracyclines can lead to permanent tooth discoloration in children under the age of 8. However, short-term administration of doxycycline or a fluoroquinolones in this age group is currently regarded as safe. Both classes of antibiotics pose potential risks to fetal health and should be avoided in pregnant women. This antibiotic prophylaxis should be administered within 2–3 days after *F. tularensis* exposure, which is the usual incubation period of tularemia. When exposure is older or the risk of infection is low, clinical and serological monitoring must be considered and antibiotic treatment administered if tularemia clinical symptoms develop. It is to be noted that recommended prophylaxis and treatment of tularemia use the same antibiotics, at the same dosage and duration. Acquired resistances to antibiotics have not been reported so far in natural strains of *F. tularensis* but have been selected *in vitro* to aminoglycosides, tetracyclines, fluoroquinolones, the macrolides, and rifampicin (Bhatnagar et al., 1994; Pavlov et al., 1996; La Scola et al., 2008; Gestin et al., 2010; Sutera et al., 2014). Antibiotic-resistant strains of *F. tularensis* could be engineered in the context of biological warfare (Alibek and Handelman, 1999; Wade, 2000).

Tularemia can be acquired during occupational or leisure activities, with similar routes and modes of infection. In tularemia endemic areas, prophylactic measures other than vaccines and antibiotics are mainly individual and should be adapted to the usual sources and modes of infection. Using protective equipment (gloves, glasses, and a respiratory mask) when manipulating game or other wildlife animals and their carcasses is of primary importance. The same precautions should be considered when manipulating sick or dead pets. Animal bites or scratches should be avoided and require considering tetanus and rabies vaccination, an antibiotic prophylaxis, and clinical monitoring when they have occurred (Ellis and Ellis, 2014). Amoxicillin/clavulanic acid is the recommended first-line prophylactic treatment after animal bites or scratches (Ellis and Ellis, 2014). However, this antibiotic is ineffective against *Francisella tularensis*, while this bacterium is susceptible to doxycycline and fluoroquinolones (Caspar and Maurin, 2017). In areas where tularemia is endemic, the potential for exposure to *F. tularensis* should be considered, particularly in situations involving bites or scratches from small rodents or other wildlife. In such cases, doxycycline or a combination of a fluoroquinolone and clindamycin, which are second-line prophylactic treatment after animal bites or scratches, would be preferred alternatives (Ellis and Ellis, 2014). Proper cooking of meat or other food products from game is essential, as is avoiding drinking unsafe water. Avoiding contact with dirty and stagnant water and wearing a mask when exposed to aerosols from terrestrial sources (especially fodder and compost) is an often neglected but important precaution in tularemia endemic areas. Finally, protective measures against tick and mosquito bites in the respective tularemia endemic areas is crucial, and protects people against other tick-borne and mosquito-borne infections (Grison et al., 2020; Lantos et al., 2021). The implementation of these prophylactic measures requires knowledge of these risks by the general population, people professionally exposed to *F. tularensis*, and the medical community. The zoonotic risk is usually well known to professionals exposed to animals while the rest of the population has often poor knowledge of zoonotic diseases, whether transmitted by wild animals, domestic animals, or pets (Spence et al., 2022; Vlaanderen et al., 2024).

Although knowledge of *F. tularensis* and tularemia has greatly increased over the past two decades, the number of annual human tularemia cases has increased in most endemic countries and new endemic areas have emerged (Jackson et al., 2012; Erdem et al., 2014; Maurin and Gyuranecz, 2016; Mínguez-González et al., 2021; Plymoth et al., 2024; Wu et al., 2024). The observed increase in tularemia incidence may be linked to qualitative or quantitative changes involving the animal reservoir, the environmental reservoir, arthropod vectors, as well as human behavioral changes, and better knowledge and reporting of tularemia by the medical community (Rydén et al., 2009; Mailles and Vaillant, 2014; Faber et al., 2018; Yeni et al., 2020; Bishop et al., 2023; Buettcher et al., 2024; Sholeh et al., 2024). The influence of climate change on zoonotic diseases has been extensively highlighted (Rupasinghe et al., 2022). Several studies have documented the potential impacts of these changes on tularemia (Rydén et al., 2009; Balci et al., 2014; Ma et al., 2020; Buhler et al., 2022). Notably, ongoing climate changes could affect the incidence and geographical distribution of tularemia through various mechanisms. Rising temperatures and humidity levels may promote the proliferation of arthropods such as ticks and mosquitoes, particularly due to increased reproductive rates, and extend their geographical range, especially at higher altitudes. These climate changes can also lead to an increase in populations of rodents and other wildlife that serve as the primary reservoir for *Francisella tularensis*, potentially due to greater access to nutritional resources. Additionally, warming surface waters and soils could significantly impact the environmental survival of *F. tularensis*. Strengthened winds may further facilitate the geographical dispersion of this bacterium. The simultaneous alterations in these various reservoirs and vectors of *F. tularensis* are likely to have a substantial impact on the epidemiology of human tularemia.

The prophylactic measures to combat tularemia must be strengthened. The general population should be better informed on the modes of contamination by *F. tularensis* and the clinical manifestations of tularemia. However, it is of primary importance to establish appropriate collective prophylactic measures in each endemic area considering the predominant modes of infection with *F. tularensis* in both humans and animals.

Controlling tularemia in the wild reservoir is difficult but monitoring the infection in small rodents and lagomorphs appears necessary. These two types of animals can be infected by numerous agents of zoonotic diseases and are even reservoirs for some bacteria, viruses and parasites (Hill and Brown, 2011). Surveillance of tularemia in lagomorphs is already carried out in certain countries via networks of hunters and veterinary laboratories (Moinet et al., 2016). Surveillance of the rodent population has shown to be useful to predict human tularemia cases in Finland (Rotejanaprasert et al., 2018). Monitoring zoonotic agents in wildlife is difficult to carry out, so field studies must consider the simultaneous detection of the most significant pathogens in terms of human and animal health in the studied geographic areas.

Studies have evaluated the prevalence of *F. tularensis* in ticks (Hubálek et al., 1997; Kreizinger et al., 2013; Bielawska-Drózd et al., 2018; Whitten et al., 2019; Esmaeili et al., 2023) or mosquitoes (Thelaus et al., 2014) using PCR-based tests. These tests must specifically target *F. tularensis*, avoiding for example detection of *Francisella*-like species in ticks (Kugeler et al., 2005; Escudero et al., 2008). A strategy employing MALDI TOF mass spectrometry has been developed to simultaneously identify arthropod species and the microorganisms they carry (Sánchez-Juanes et al., 2022; Jumpertz et al., 2023). This technique should be evaluated for *F. tularensis*. Prevention and control measures against diseases transmitted by arthropod vectors must include *F. tularensis* in tularemia endemic areas. Monitoring the prevalence of *F. tularensis* infection in ticks and mosquitos should be carried out regularly in known tularemia endemic areas. As previously mentioned, field studies are tedious and must be carried out considering all the major pathogens transmitted by ticks or mosquitoes in a specific geographical area.

Controlling the tick population, particularly host-seeking ticks, in a specific area seems a tedious task (Eisen and Stafford, 2021). However, controlling *F. tularensis* infection rates in ticks is even more problematic because of our inability to control the wide animal reservoir of this pathogen from which ticks become infected. The use of synthetic acaricides is highly effective to control tick populations, while natural product-based acaricides and biological agents (e.g., entomopathogenic fungi) are less often used (Eisen and Stafford, 2021). In rural tularemia endemic areas, synthetic acaricides can be used to treat the surroundings of houses combined with vegetation management. The use of synthetic acaricides in large geographic areas is not recommended due to potential deleterious effects on the environment and beneficial insects (e.g., pollinators) and a risk of development of resistance to acaricides in ticks (Eisen and Stafford, 2021).

The reasons why mosquito-borne tularemia is limited to specific areas in a few northern countries, notably Sweden and Finland, remain unknown. Tularemia outbreaks occur in these countries due to the large number of people exposed to the mosquito bites. There is no indication that this ecological situation cannot be established in other regions or countries, including outside the current tularemia endemic areas. This would represent a major public health concern since the extension of mosquito-borne tularemia areas would lead to a significant increase in epidemic cases of human tularemia. Current mosquito-control large-scale strategies include the use of insecticides and genetic biocontrol methods (Weng et al., 2024). Although effective, insecticides have deleterious effects on the environment and target too many insect species. Genetic biocontrol methods of mosquito populations include the release into the environment of massive numbers of irradiated sterile males, *Wolbachia*-infected males, or genetically modified mosquitoes expressing a lethal gene (Weng et al., 2024).

It seems crucial to monitor the aquatic reservoir of *F. tularensis* in areas where tularemia is highly endemic. Tools for specific detection of *F. tularensis* in the environment have been developed (Kaysser et al., 2008; Broman et al., 2011; Simşek et al., 2012; Janse et al., 2018; Brunet et al., 2021). The microbial water surface quality has been established and is monitored in many countries at least for enteric pathogens (Islam et al., 2021). Modern methods have simplified this surveillance (Bonadonna et al., 2019). In endemic areas it would be useful to add monitoring of certain zoonotic agents which have a water reservoir such as *F. tularensis* and *Leptospira* species. Monitoring the prevalence of *F. tularensis* in its various reservoirs is essential for combating both animal and human cases of tularemia. This surveillance can also help identify changes in the epidemiology of the disease that may arise due to factors such as climate variations, shifts in the population density and geographic distribution of wild animal reservoirs, alterations in arthropod vector populations, and emerging human practices.

## 13 Conclusion

Tularemia is a disease whose incidence is gradually increasing in most endemic countries. No tularemia vaccination is currently approved for use in humans or animals. Antibiotic prophylaxis is recommended only for individuals with a strong suspicion of exposure to *F. tularensis*, such as contact with a culture of this pathogen. Current prophylactic measures against *F. tularensis* are primarily individual and focus on various infection sources. Understanding the primary modes of infection in endemic areas is essential for effective prevention strategies. Collective measures should include monitoring *F. tularensis* prevalence in key reservoirs such as wild lagomorphs, small rodents, arthropod vectors, and aquatic environments. This “One Health” approach is challenging to implement but essential to control the incidence and geographic distribution of tularemia in humans and animals, and to detect any major changes in the epidemiology of this disease. The potential spread of mosquito-borne tularemia beyond its current endemic regions could have significant public health consequences.
